# Asymptomatic intracranial aneurysms in beta-thalassemia: a three-year follow-up report

**DOI:** 10.1186/s13023-020-1302-3

**Published:** 2020-01-20

**Authors:** Renzo Manara, Martina Caiazza, Rosanna Di Concilio, Angela Ciancio, Elisa De Michele, Caterina Maietta, Daniela Capalbo, Camilla Russo, Domenico Roberti, Maddalena Casale, Andrea Elefante, Fabrizio Esposito, Sara Ponticorvo, Andrea Gerardo Russo, Antonietta Canna, Mario Cirillo, Silverio Perrotta, Immacolata Tartaglione

**Affiliations:** 1 Dipartimento di Medicina, Chirurgia e Odontoiatria, Scuola Medica Salernitana, Sezione di Neuroscienze, Università di Salerno, Salerno, Italy; 2 Dipartimento della Donna, del Bambino e di Chirurgia Generale e Specialistica, Università degli studi della Campania “Luigi Vanvitelli”, Via L. De Crecchio 4, Naples, Italy; 3 Dipartimento di Pediatria, Ospedale “Umberto I”, Nocera Inferiore, Italy; 4 Unità Operativa Ematologia - Day Hospital di Talassemia, Ospedale “Madonna delle Grazie”, Matera, Italy; 5 Medicina Trasfusionale AUO “San Giovanni di Dio e Ruggi D’Aragona”, Salerno, Italy; 6 Neuroradiologia, Università degli Studi di Napoli “Federico II”, Naples, Italy; 7 Dipartimento di Scienze Mediche, Chirurgiche, Neurologiche, Metaboliche e dell’Invecchiamento, Università della Campania “Luigi Vanvitelli”, Naples, Italy

**Keywords:** Aneurysm, Beta-thalassemia, Magnetic resonance angiography, Subarachnoid hemorrhage

## Abstract

**Background:**

No information is currently available regarding the natural history of asymptomatic intracranial aneurysms in beta-thalassemia, raising several concerns about their proper management.

**Methods:**

We performed a prospective longitudinal three-year-long MR-angiography study on nine beta-thalassemia patients (mean-age 40.3 ± 7.5, six females, 8 transfusion dependent) harboring ten asymptomatic intracranial aneurysms. In addition, we analyzed the clinical files of all adult beta-thalassemia patients (160 patients including those followed with MR-angiography, 121 transfusion dependent) referring to our Centers between 2014 and 2019 searching for history of subarachnoid hemorrhage or history of symptomatic intracranial aneurysms.

**Results:**

At the end of the three-year-long follow-up, no patient showed any change in the size and shape of the aneurysms, none presented new intracranial aneurysms or artery stenoses, none showed new brain vascular-like parenchymal lesions or enlargement of the preexisting ones. Besides, in our database of all adult beta-thalassemia patients, no one had history of subarachnoid hemorrhage or history of symptomatic intracranial aneurysms.

**Conclusions:**

Incidental asymptomatic intracranial aneurysms do not seem to be associated, in beta-thalassemia, with an increased risk of complications (enlargement or rupture) at least in the short term period, helping to optimize human and economic resources and patient compliance during their complex long-lasting management.

## Introduction

Incidental asymptomatic intracranial saccular aneurysms raise several management concerns as they might either carry a benign course or turn out into subarachnoid hemorrhage, with associated potentially significant morbidity and mortality [1]. Once detected, a tailored assessment of risk of rupture versus risk of intervening is needed to determine the best option for each patient. This includes the evaluation of demographic/clinical data and aneurysm features (mainly aneurysm site and size) that are usually considered in a global score (e.g. the PHASES score, see online material). High scores prompt for aneurysm clipping or endovascular treatment because of the high risk of aneurysm rupture. Low scores suggest a less aggressive approach with, for example, a 3–5 year imaging monitoring of aneurysm growth as growing aneurysms are associated with a high risk of rupture [2]. Once aneurysm stability has been established, reduction in imaging frequency is considered reasonable [1]. However, there are no guidelines regarding the optimal timing for serially imaging these aneurysms especially when specific genetically determined conditions coexist.

Beta-thalassemia is a heterogeneous group of inherited disorders characterized by defective production of the beta chain of hemoglobin. This condition has been repeatedly associated to early brain cerebrovascular involvement [3, 4] because of multiple coexisting disease-related putative risk factors of cumulative neural tissue injury [5, 6]. In addition, beta-thalassemia patients often undergo splenectomy that has been associated, at least in non-transfusion dependent beta-thalassemia (NTDT) patients, to an increased rate of intracranial artery stenoses and aneurysms [7]. The latter have been found in 5/29 (17.2%) splenectomized NTDT adults in the first observational transverse MR-angiography study [7]. However, this finding was not confirmed by subsequent case-control studies which reported no cases of intracranial aneurysms in 80 thalassemia patients (transfusion dependent, NTDT and E-beta-thalassemia patients) [8] or no significantly increased incidence rate in 73 transfusion dependent and NTDT beta-thalassemia patients compared to healthy controls (9.3% vs 8.9%, respectively) [9]. Beside the unsolved issue of an increased incidence rate of intracranial aneurysms, no information is currently available regarding their natural course, whenever they are incidentally discovered. While symptomatic or high PHASES score aneurysms would likely undergo prompt endovascular or surgical treatment, so far, the proper management of small incidental asymptomatic aneurysms in beta-thalassemia patients remains unknown.

For this reason, we investigated beta-thalassemia patients harboring asymptomatic intracranial aneurysms referring to our Centers by means of non-invasive MR-angiography three years after aneurysm detection thus providing the first report of their natural course. In addition, we reviewed the current literature and analyzed the clinical files of all adult beta-thalassemia patients referring to our Centers in the last 5 years searching for history of subarachnoid hemorrhage or history of symptomatic intracranial aneurysms.

## Material and methods

### Study population

Nine patients (mean-age 40.3 ± 7.5, age-range 26–49, 6/9 females, 8/9 TDT; see Additional file 1: Table S1) referring to our four Centers in South Italy (Napoli, Salerno, Nocera Inferiore, Matera) were found to harbor asymptomatic intracranial aneurysms and were considered in this follow-up study. No beta-thalassemia patient underwent any specific treatment as aneurysms were all asymptomatic, relatively small (range 2-5 mm) and had imaging and clinical features leading to a very low PHASES score (all had PHASES score = 0 but a patient harboring a distal middle cerebral artery aneurysm receiving a score of 2) generally associated with low risk of rupture. One patient carried a successful pregnancy between the first MR-angiography and the subsequent MR follow-up.

We investigated the clinical files of all the adult beta-thalassemia patients followed in the last 5 years (2014–2019) in the above mentioned referral Centers (160 patients, 121/160 TDT) searching for history of cerebral aneurysm complications.

### MR-angiography

All patients underwent non-invasive aneurysm diagnosis on the same 3 T MR-scanner (MAGNETOM Skyra, Siemens, Erlangen Germany) by means of 3D-multi-slab Time of Flight sequence (TR/TE 21/3.43 ms; voxel-size 0.6*0.6*0.7 mm; field of view 200 mm; numbers of partitions 120; number of partitions/slab 40; acquisition-time 3 min 34 s) [9].

After three years, eight patients had MR-angiography on the same MR scanner with the same sequence, while one had follow-up MR-angiography on a different 1.5 T MR-scanner.

Both partition images and 3D maximum intensity projection reconstructions were evaluated by the same neuroradiologist (RM) with experience in cerebrovascular and intracranial vascular disorders.

The study protocol also included a 3D fluid attenuated inversion recovery (FLAIR, TR/TE/TI 5000/387/1800 ms; voxel-size 1*1*1 mm; echo-train length 278; field of view 230; acquisition-time 4 min 32 s). Axial, coronal and sagittal multiplanar reconstructions of the whole brain were obtained (slice thickness 3 mm without interslice gap).

### Literature review

All published studies dealing with intracranial aneurysms or subarachnoid hemorrhage in beta thalassemia were searched in www.pubmed.com and www.google.com using “beta-thalassemia” and “intracranial aneurysm” or “subarachnoid hemorrhage”. The retrieved articles were analyzed and non-pertinent studies were excluded. The search was thereafter extended to the references of the identified papers.

The study protocol was approved by the local Ethical Committee and contrast medium was not used for MR-angiography. All patients gave their written informed consent.

## Results

At the 3-year MRI and MR-angiography follow-up, no patient showed any change in the size and shape of the aneurysms, none presented new intracranial aneurysms or artery stenoses, none showed new white matter lesions or enlargement of the preexisting ones (see Additional file 1: Table S1).

Besides, in our database of all adult beta-thalassemia patients referring to our Centers in the last 5 years no patient had history of subarachnoid hemorrhage or history of symptomatic intracranial aneurysms.

## Discussion

This longitudinal MR-angiography study shares our experience regarding the natural history of asymptomatic incidental intracranial aneurysms in beta-thalassemia. According to our findings, intracranial aneurysms with low PHASES scores (≤2) might be safely handled conservatively also in beta-thalassemia patients. In fact, none of 10 intracranial artery aneurysms (nine beta-thalassemia patients) followed-up during the study showed any change in size and shape in the three years after their detection. In addition, none among the 160 adult beta-thalassemia patients referring to our Centers in South Italy had history of previous subarachnoid hemorrhage, i.e. the most common and feared complication of intracranial aneurysms. Therefore, Italian beta-thalassemia patients treated according to the current guidelines not only do not seem to have an increased risk of cerebrovascular changes including intracranial aneurysms, [9, 10] but also, when the latter are detected incidentally, they do not seem to present signs of short-term evolution.

The literature review about intracranial aneurysms in beta-thalassemia reveals that data are strikingly scarce and controversial. Even though beta-thalassemia patients had shown a high rate of intracranial aneurysms (17.2%) [7] the study lacked a control group, it was not confirmed by subsequent larger case-control studies, [8, 9] and it considered a subgroup of patients (NTDT patients) undergoing a specific surgical procedure (splenectomy) that is nowadays more and more delayed or even prevented. Therefore, the current literature leaves still unsolved whether such a high rate of intracranial aneurysms were limited to environmental, treatment or genetic factors of a specific world region or it truly unveiled a disease-related vascular vulnerability of adult beta-thalassemia patients. Indeed, a few cases of subarachnoid hemorrhage, [11–13] have been already reported in beta-thalassemia, apparently supporting the suspicion of an increased aneurysm-related jeopardy. However, we found no cases of subarachnoid hemorrhage in the clinical life-long history of our whole adult beta-thalassemia population, suggesting that its occurrence is at least not so common when investigated systematically. In addition, none of the beta-thalassemia patients with subarachnoid hemorrhage reported in the literature had angiographic evidence of intracranial aneurysm as the cause of intracranial bleeding (two of them had moyamoya disease while the third patient had normal angiography findings).

### Limits of the study

As reported in the Table 1, one patient performed autonomously the follow-up MR-angiography with a 1.5 T MR-scanner, i.e. with a scanner different than that used for aneurysm detection three years before and different than that used for all other study patients. However, MR-angiography is a robust sequence and the quality of the 1.5 T MR-angiography was preliminarily judged adequate for the study purposes before its inclusion in the analysis. Considering the rarity of beta thalassemia condition, its even rarer coexistence with intracranial aneurysm and the lack of information on this issue in the literature, the exclusion of this patient would have omitted precious data on the natural history of incidentally detected intracranial aneurysm in beta thalassemia. In general, the limited sample size of both the whole group of beta-thalassemia patients and the subgroup harboring intracranial aneurysms represents the main limit of this study. However, this is the first report on the follow-up of asymptomatic intracranial aneurysms in beta-thalassemia and the first investigation (including an exhaustive literature search) on intracranial aneurysm complications in beta-thalassemia. Even though this report cannot be conclusive, it represents so far the sole reference for the management of incidentally detected intracranial aneurysms in a beta-thalassemia patient.

**Table 1:** Characteristics of beta-thalassemia patients harboring intracranial aneurysms; intracranial aneurysm main features at diagnosis and at 3-year MR-angiography follow-up

Hemoglobin genotype	Age^a^ (years)	Sex	βT-Ph	Size (mm)	Side	Splen.	Aneurysm location(s)	Aneurysm treatment	MRA follow-up (3 years)
β0/β+	26	F	NTDT	2 mm	R	Yes	carotid-ophthalmic	Observation	Unchanged
unknown	41	F	TDT	2 mm	L	Yes	carotid-ophthalmic	Observation	Unchanged
unknown	40	F	TDT	4 mm	R	No	carotid-ophthalmic	Observation	Unchanged
unknown	34	F	TDT	4 mm	L	Yes	carotid-ophthalmic	Observation	Unchanged
β0/ααα	43	M	TDT	5 mm	L	Yes	distal M1	Observation	Unchanged
unknown	44	M	TDT	5 mm	R	Yes	carotid-ophthalmic	Observation	Unchanged
				2 mm	L		carotid siphon		Unchanged
β0/β0	49	M	TDT	2 mm	L	Yes	carotid siphon	Observation	Unchanged
β0/β0	45	F	TDT	3 mm	L	Yes	carotid siphon	Observation	Unchanged°
unknown	46	F	TDT	3 mm	L	Yes	carotid siphon	Observation	Unchanged

*F* female, *M* male, *βT-Ph* beta-thalassemia phenotype, *NTDT* non transfusion dependent thalassemia, *TDT* transfusion dependent thalassemia, *R* right, *L* left, *M1* middle cerebral artery main trunk, ° MRA follow-up performed with a 1.5 T scanner, ^a^age at diagnosis

In conclusion, it is unclear whether beta-thalassemia patients have an increased rate of intracranial aneurysms and there seems to be no evidence that incidentally detected asymptomatic intracranial aneurysms in beta-thalassemia are associated with an increased risk of complications compared to the normal population at least in the short term period.

Future studies will clarify whether beta-thalassemia should be considered at increased cerebrovascular risk in the medium- and long-term follow-up, helping to optimize human and economic resources and patient compliance in their complex long-lasting management.

## Supplementary information

**Additional file 1: Table S1.** Summary of PHASES score (from Nasr DM et al. [1]).

## Data Availability

The datasets during and/or analysed during the current study available from the corresponding author on reasonable request.
