# Supplementary material for: Asymptomatic intracranial aneurysms in beta-thalassemia: a three-year follow-up report
Source: Orphanet J Rare Dis. 2020 Jan 20;15:21. doi: 10.1186/s13023-020-1302-3 (PMC6971974; doi:10.1186/s13023-020-1302-3)
Supplement: Supplementary file 1 — Additional file 1: Table S1. Summary of PHASES score (from Nasr DM et al. [1]). [file 13023_2020_1302_MOESM1_ESM.docx]

Additional file 1: Table S1. Summary of PHASES score (from Nasr DM et al., 2016)

|  | Number of points |
| --- | --- |
| *Population*  North America or European (not Finnish)  Japanese  Finnish | 0  3  5 |
| *Hypertension*  No  Yes | 0  1 |
| *Age*  <70 years  ≥70 | 0  1 |
| *Size of aneurysm*  <7 mm  7–9.9 mm  10–19.9 mm  ≥20 mm | 0  3  6  10 |
| *Earlier SAH from another aneurysm*  No  Yes | 0  1 |
| *Site of aneurysm*  ICA  MCA  ACA/Pcom/posterior | 0  2  4 |

Five-year rupture risk ranges from 0.4 % for ≤2 points to 17.8 % for ≥12 points
